# Altered Spontaneous Regional Brain Activity in the Insula and Visual Areas of Professional Traditional Chinese Pingju Opera Actors

**DOI:** 10.3389/fnins.2018.00450

**Published:** 2018-07-03

**Authors:** Weitao Zhang, Fangshi Zhao, Wen Qin, Lin Ma

**Affiliations:** ^1^Department of Radiology, People’s Liberation Army General Hospital, Beijing, China; ^2^Department of Radiology, Tianjin Medical University General Hospital, Tianjin, China

**Keywords:** resting-state fMRI, ALFF, ReHo, Pingju opera actor, neural efficiency

## Abstract

Recent resting-state fMRI studies have revealed neuroplastic alterations after long-term training. However, the neuroplastic changes that occur in professional traditional Chinese Pingju opera actors remain unclear. Twenty professional traditional Chinese Pingju opera actors and 20 age-, sex-, and handedness-matched laymen were recruited. Resting-state fMRI was obtained by using an echo-planar imaging sequence, and two metrics, amplitude of low frequency fluctuation (ALFF) and regional homogeneity (ReHo), were utilized to assess spontaneous neural activity during resting state. Our results demonstrated that compared with laymen, professional traditional Chinese Pingju actors exhibited significantly decreased ALFF in the bilateral calcarine gyrus and cuneus; decreased ReHo in the bilateral superior occipital and calcarine gyri, cuneus, and right middle occipital gyrus; and increased ReHo in the left anterior insula. In addition, no significant association was found between spontaneous neural activity and Pingju opera training duration. Overall, the changes observed in spontaneous brain activity in professional traditional Chinese Pingju opera actors may indicate their superior performance of multidimensional professional skills, such as music and face perception, dancing, and emotional representation.

## Introduction

Understanding the neuroplastic changes that occur when training for a series of skills has significant implications on health and society. Scientific reports assessing neuroplastic changes have demonstrated that certain practices aid in the recovery of patients with brain damage or neurodegeneration (Fan et al., 2015; Pedersen et al., 2016; Fluet et al., 2017), and training-induced neuroplastic alterations have become a hot topic in modern neuroscience.

Imaging techniques have greatly furthered the knowledge of neuroplastic changes, and substantially improved our knowledge of neuroplastic changes (Alain et al., 2015; Bar and DeSouza, 2016; Vaquero et al., 2016; Ji et al., 2017). Previous studies assessing brain neuroplasticity in expertise models, including musicians (Harris and de Jong, 2015), athletes (Kellar et al., 2018), and actors (Etzel et al., 2016) and acupuncturists (Dong et al., 2013), demonstrated that specialized training modulates brain response patterns using task-state functional MRI. Recently, researchers recognized that neuroplasticity in response to long-term training might also be detectable in the resting state (Tanaka and Kirino, 2016; Tao et al., 2017). Resting-state functional MRI (rs-fMRI), which is a non-invasive method for assessing regional and neural circuitry function at rest, is a promising tool to investigate the human brain (Yuan et al., 2016, 2017; Liu et al., 2017). This method requires minimal patient compliance, avoids potential performance confounders associated with the cognitive activation paradigms in task-design fMRI research, and is relatively easy to implement in clinical studies (Liu et al., 2015; Wang et al., 2016; Dierker et al., 2017). Spontaneous low-frequency fluctuations detected by rs-fMRI are significantly associated with the maintenance of ongoing, internal representations that may be related to prior experience (Lewis et al., 2009). Therefore, rs-fMRI has been increasingly utilized in research investigating brain neuroplasticity. Some studies on neuroplasticity showed changed resting-state functional (rs-FC) connectivity strength/patterns in expertise models (Tanaka and Kirino, 2016; Tao et al., 2017). However, whether the baseline brain activity was changed is unknown, and this is a fundamental issue.

In contrast to rs-FC, which measures the synchronization between remote brain regions, ALFF and ReHo are two widely used methods used to explore local spontaneous brain activity (Guo et al., 2011; Liu et al., 2013; Ji et al., 2017). ALFF is used to measure regional brain activity by computing the square root of the power spectrum in the low-frequency range (Zang et al., 2007a), and ReHo is applied to evaluate the synchronization between the spontaneous activity of a given voxel and its nearest neighboring voxels (usually 26 voxels) (Zang et al., 2007b). To date, several studies have used these two measures to examine experience- or training-related neuroplasticity in the human brain. For example, a previous study found that decreased ALFF in the left superior parietal lobe in professional badminton players is induced by specialized badminton practice and training (Di et al., 2012). One study revealed abnormal ALFF in vision and vision-related regions in patients with late monocular blindness (Li et al., 2016). Another study found that ReHo in the occipital cortex is increased in people with early-onset blindness, which might be explained by experience-related neuroplasticity (Liu et al., 2011). Decreased ReHo has been demonstrated in prefrontal regions, whereas ReHo increased in the posterior cingulate cortex and insula of long-term heavy male smokers (Yu et al., 2013). However, previous studies reported that insula-based networks in professional musicians are reorganized by musical training (Zamorano et al., 2017) and that neuroanatomical alterations develop in professional gymnasts’ brains (Wang et al., 2013). Studies on brain activity alterations in opera actors are relatively limited compared with those in musicians and athletes. Therefore, we proposed that professional traditional Pingju opera actors, who undergo long-term specialized training from childhood, represent ideal individuals for investigating neuroplastic changes. Pingju opera is a popular traditional Chinese opera with large audiences in northern China. Professional traditional Chinese Pingju opera actors play core roles on the Pingju opera stage. They integrate song, speech and dance skills with movements that are suggestive and symbolic, rather than realistic, and every movement must be performed in time with the music. All these performing skills include three interacting components: (1) exceptional voice processing ability for singing and dialog, which enable professional Pingju actors attract the attention of the audience; (2) flexibility and coordination of the body to convey different characters and communicate with other Pingju actors on the stage; and (3) emotional regulation ability allowing the inner world of characters to be experienced and enabling the professional Pingju actor to express characters’ feelings of happiness, anger, surprise, and sorrow. Therefore, professional Pingju opera actors are novel and robust expertise models.

In this study, we combined ALFF and ReHo approaches to investigate neuroplasticity using the expertise model of professional traditional Chinese Pingju actors. First, based on previous studies showing that local spontaneous brain activity is altered by prior training experience, we expected alterations in the resting-state activity of professional Pingju opera actors in regions responsible for voice processing, coordination and flexibility, and higher order emotional control. Second, correlation analyses were also conducted between both the ALFF and ReHo of areas, showing significant between-group differences and the duration of Pingju opera training.

Given the paucity of reports related to traditional Chinese Pingju opera, this study provides a novel connection between spontaneous brain activity and neuroplasticity.

## Materials and Methods

### Participants

Twenty professional right-handed adult professional traditional Chinese Pingju opera actors were recruited from Tianjin Pingju Opera Theater and Tianjin Baipai Pingju Opera Theater. All actors had been professionally trained since 6–7 years of age and with an average professional performance experience of 23.75 ± 2.47 years (range: 20–32 years). In addition, 20 age-, gender-, handedness, and education-matched laymen were recruited. Laymen with musical professional education experience were excluded from this study. All 40 volunteers were healthy subjects without any history of neurological or psychiatric disease. There were also no major neurological or psychiatric illnesses among their first-degree relatives.

This study was approved by the Ethics Committee of Tianjin Medical University General Hospital. Written informed consent was obtained from each subject after the subjects were provided a detailed description of the study.

### Imaging Data Acquisition

MRI data were acquired using a 3.0-Tesla MR system (Discovery MR750, General Electric, Milwaukee, WI, United States). Tight foam paddings were used to minimize head motion, and earplugs were used to reduce reactions induced by noise. During fMRI scanning, the participants were required to remain still, relax their minds, and stay awake. Resting-state fMRI data were acquired using a single-shot echo-planar sequence with the following parameters: repetition time (TR)/echo time (TE) = 2000/45 ms, field of view (FOV) = 220 mm × 220 mm, matrix = 64 × 64, flip angle = 90°, slice thickness = 4 mm, gap = 0.5 mm. For each participant, the brain volume comprised 32 axial slices, and each functional run contained 180 image volumes. In addition, T1-weighted 3D images were also obtained using a brain volume sequence with the following parameters: TR/TE/inversion time (TI) = 8.17/3.18/450 ms, FOV = 256 mm × 256 mm, matrix = 256 × 256, slice thickness = 1 ms, slice number = 188.

### Data Preprocessing

Resting-state fMRI scans were preprocessed using the SPM8 software package^1^ and DPARSF V2.3 software^2^. After removing the first 10 images to allow the signal to reach equilibrium, slice timing was performed to correct for the temporal differences between slices. Then, rigid body realignment was carried out to evaluate and correct head motion. All subjects’ fMRI data were within the predefined head motion thresholds (translational and rotational motion parameters less than 2 mm and 2°, respectively). We also calculated the frame-wise displacement (FD) and compared the differences in mean FD between the actor and NC groups (two-sample *t*-test, *p* < 0.05) (Van Dijk et al., 2012). A two-step coregistration method was used to transform the regressed fMRI data into the MNI space. First, the mean realigned fMRI images were linearly (6-parameters) coregistered with individual structural images; then, the structural images were segmented into gray matter, white matter and cerebral spinal fluid (CSF), and were then non-linearly coregistered with the standard MNI T1-weighted template using the Diffeomorphic Anatomic Registration Through Exponentiated Lie algebra (DARTEL method) (Ashburner, 2007). The non-linear parameters were applied to the fMRI data to normalize the data to the MNI space and resliced with a 3 mm × 3 mm × 3 mm voxel size. There were some differences between the preprocessing pipelines for ALFF and ReHo in the subsequent steps. To calculate ALFF, the normalized fMRI data were smoothed with a 6 mm × 6 mm × 6 mm full-width at half maximum (FWHM) Gaussian kernel. Then, several nuisance covariates, including the 24-parameter head motion (6 head motion parameters, 6 head motion parameters one time point before, and the 12 corresponding squared items), the average BOLD signals of the CSF and white matter, and the spike time points with FD > 0.5 were regressed out from the data. The preprocessed fMRI data were then used in the ALFF calculation. To calculate ReHo, after normalization, the fMRI data underwent nuisance regression as described above, and were then band pass filtered with a frequency range of 0.01–0.08 Hz. Finally, the preprocessed data were used for the ReHo calculation.

### ALFF Calculation

The time series were transformed to frequency domains using fast Fourier transform, and the power spectrum was obtained. The square root was calculated at each frequency of the power spectrum, and the averaged square root was obtained across 0.01–0.08 Hz at each voxel (Lewis et al., 2009; Liu et al., 2017). This averaged square root was taken as the ALFF, and the ALFF of each voxel was divided by the global mean ALFF value.

### ReHo Calculation

Individual ReHo maps were obtained by calculating the Kendall correlation coefficient of a given voxel and those of its neighboring voxels (26 voxels) in a voxel-wise manner (Zang et al., 2007b). Each ReHo map was also scaled by its global mean, and finally smoothed with a 6 mm FWHM Gaussian kernel.

### Statistical Analysis

The group differences in age and head motion (mean FD) were evaluated using two-sample *t*-tests (*p* < 0.05). The group difference in gender was carried out using the chi-square test (*p* < 0.05). Voxel-wise two-sample *t*-tests were performed using SPM8 to compare the differences in scaled ALFF and ReHo between the opera actors and normal controls. Cluster-wise family-wise error (FWE) corrections were applied to control for multiple comparisons with a voxel-wise uncorrected threshold of *p* < 0.001 and a cluster-wise corrected threshold of *p* < 0.05. Furthermore, voxel-wise Pearson correlation analyses were performed to explore the relationships between the alteration in brain regional activity (ALFF and ReHo) and the duration of opera training in the actor group (uncorrected threshold of *p* < 0.001 and cluster-wise FWE correction of *p* < 0.05).

## Results

### Demographics of the Participants

The demographic data are summarized in **Table 1**. There were no significant differences in age (*t* = 0.394, *p* = 0.534), gender (χ^2^ = 0, *p* = 1), or mean FD (*t* = 3.158, *p* = 0.084), which demonstrates that age, gender, and head motion cannot explain any possible differences in ALFF and ReHo between Pingju opera actors and laymen.

**Table 1:** Demographic information of the involved professional Pingju actors and laymen.

Group	*n*	Gender	Age (years)	Mean FD	Years of training
Actors	20	10/10	30.00 ± 2.43	0.023 ± 0.014	23.75 ± 2.47
Laymen	20	10/10	29.10 ± 5.94	0.016 ± 0.011	/
Statistics		0	0.394	3.158	/
*p*		1	0.534	0.084	/


### ALFF Alterations in Pingju Opera Actors and the Association With Duration of Training

**FIGURE 1:**
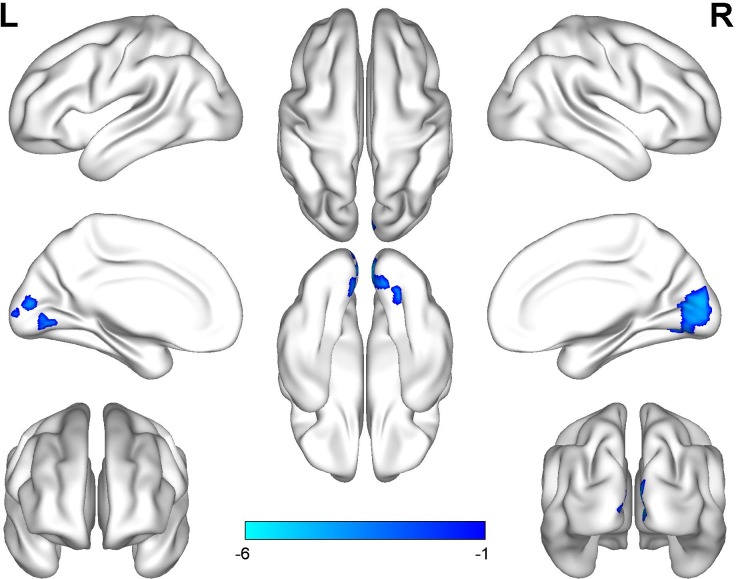
Group differences in ALFF between Pingju opera actors and laymen. Two-sample *t*-tests were performed with cluster-wise multiple comparison correction (FWE, *p* < 0.05). The color bar represents the *t*-values.

The professional traditional Chinese Pingju opera actors generally had significantly lower ALFF in the bilateral calcarine gyrus and the cuneus (**Figure 1** and **Table 2**) than the laymen (*p* < 0.05, cluster-wise FWE correction). We did not observe any significant association between regional ALFF and duration of opera training (*p* < 0.05, cluster-wise FWE correction).

**Table 2:** Regions of altered ALFF in professional Pingju actors.

Brain regions	BA	No. voxels	Peak MNI coordinates			Peak *T*-value
			*x*	*y*	*z*	
Bilateral calcarine gyrus	17/18	311	3	–87	12	4.70


*ALFF, amplitude of low-frequency fluctuation; BA, Brodmann Area; MNI, Montreal Neurological Institute.*

### ReHo Alterations in Pingju Opera Actors and the Association With Duration of Training

**FIGURE 2:**
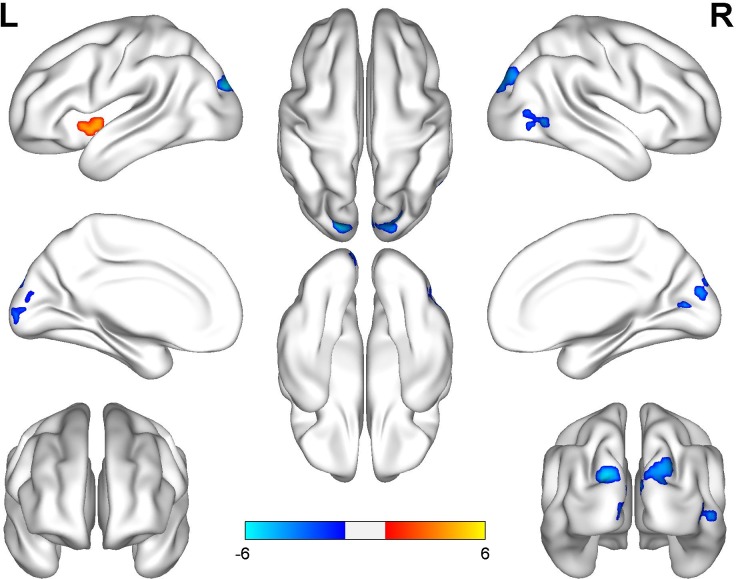
Group differences in ReHo between Pingju opera actors and laymen. Two-sample *t*-tests were performed with cluster-wise multiple comparison correction (FWE, *p* < 0.05). The color bar represents the *t*-values.

The professional traditional Chinese Pingju opera actors generally had significantly higher ReHo in the left anterior insula and significantly lower ReHo in the bilateral superior occipital and calcarine gyri, the cuneus, and the right middle occipital gyrus (**Figure 2** and **Table 3**) than the laymen (*p* < 0.05, cluster-wise FWE correction). There was also no significant relationship between regional ReHo and duration of opera training (*p* < 0.05, cluster-wise FWE correction).

**Table 3:** Regions of altered ReHo in professional Pingju actors.

Brain regions	BA	No. voxels	Peak MNI coordinates			Peak *T*-value
			*x*	*y*	*z*	
Bilateral SOG/CalG/cuneus	17/18/19	314	–18	–87	24	–5.91
Right MOG	19	64	54	–72	0	–4.77
Left AI	–	70	–36	3	–3	4.42


*ReHo, regional homogeneity; BA, Brodmann Area; MNI, Montreal Neurological Institute; SOG, superior occipital gyrus; CalG, calcarine gyrus; MOG, middle occipital gyrus; AI, anterior insular cortex.*

## Discussion

To the best of our knowledge, this is the first study using the ALFF and ReHo methods to detect regional spontaneous brain activity in professional traditional Chinese Pingju opera actors. A decreased ALFF of the bilateral calcarine gyrus and cuneus were found in professional Pingju actors. In addition, professional traditional Chinese Pingju opera actors exhibited significantly higher ReHo than did laymen in the left anterior insula and significantly lower ReHo in the bilateral occipital gyrus, including the bilateral superior occipital and calcarine gyri, the cuneus, and the right middle occipital gyrus.

In this study, we combined both ALFF and ReHo methods to analyze spontaneous low-frequency fluctuations in cortices (Balduzzi et al., 2008), which play a significant role in maintaining ongoing, internal representations for neuroplasticity (Lewis et al., 2009). ALFF and ReHo are biologically different metrics. ALFF is a reproducible and reliable approach to detect baseline brain activity in healthy volunteers (Zuo et al., 2010; Dong et al., 2015) and patients (Guo et al., 2012). Some studies have shown changes in baseline brain activity in expertise models (Di et al., 2012; Dong et al., 2015). In contrast, ReHo is applied to evaluate the synchronization between the spontaneous activity of a given voxel and its nearest neighboring voxels (usually 26 voxels) (Zang et al., 2007b). ReHo is the most widely used, efficient and reliable index representing local FC (Zuo and Xing, 2014; Jiang and Zuo, 2016). ReHo is sensitive to differences in spontaneous activity between groups and conditions (Liu et al., 2010). Several researchers observed altered ReHo brain activity in experts (Dong et al., 2014) and patients (Zhang et al., 2012). ReHo is considered complementary to ALFF. Therefore, elaborated neuroplasticity in professional Pingju opera actors can be detected using both the ALFF and ReHo methods.

Our study showed an increased coherence of regional BOLD signal fluctuations in the left anterior insula. This area is responsible for disparate affective, cognitive, olfactory, auditory, visual, and musical information (Kurth et al., 2010; Klabunde et al., 2017; Zamorano et al., 2017). The left anterior insula has both speech and language processing ability, which is attributed to its direct anatomical connection to the inferior and lateral frontal areas (Jakab et al., 2012). Further, the left anterior insula has multiple functional connections with frontal regions involved in language processing, such as the frontal operculum and the prefrontal cortex (Augustine, 1996). Based on the above observations, we suggest that this region might contribute to voice processing ability in professional Pingju opera actors.

Numerous neuropsychological and functional neuroimaging research has found that the anterior insula plays a pivotal role in emotional processing (Lamm and Singer, 2010; Seara-Cardoso et al., 2016; Smith et al., 2017). Professional traditional Chinese Pingju opera actors depict different emotions, such as happiness, anger, surprise, and sorrow, using graceful voice and body language, all of which place an enormous demand on the insula. One study found increased brain activity in the anterior insula of individuals with compassion and empathy training experience (Klimecki et al., 2014). Another study described improved emotional symptoms and increased ReHo values for the anterior insula in ischemic stroke patients that received acupuncture treatment (Ping et al., 2017). Meanwhile, several studies demonstrated increased left anterior insula activation in individuals experiencing the emotions of others (Singer et al., 2004; Caria et al., 2010; Duerden et al., 2013; Kann et al., 2016). Therefore, we suggested that the left anterior insula might be responsible for emotional regulation in professional Pingju opera actors.

In this study, brain regions with decreased ALFF and ReHo signal commonly included the primary visual cortex (Wurm et al., 2017), which is related to the initial processing of visual information, and the dorsal visual pathways, which are specialized for spatial processing (Kujovic et al., 2012). One possible explanation is the “neural efficiency theory,” which hypothesizes that decreased ALFF and ReHo values in professional Pingju opera actors might be induced by the increased efficiency of visual functions. Thus, professional Pingju opera actors might require a lower threshold to process visual information than do laymen. Previous studies also found improved neural efficiency in the visual regions of training/expertise models (Motes et al., 2008; Zimmer et al., 2012; Pang et al., 2013; Guo et al., 2017). One study describes that decseased ALFF in visual areas is associated with superior Chinese reading abilities (Qian et al., 2016).

Therefore, we predicted that the superior lingual skills of Pingju opera actors might be related to decreased ALFF in visual areas. Another study detected decreased ReHo in visual regions in people with internet gaming addictopn, which suggested that neuroplastic alterations in visual regions might generate enhanced sensory-motor coordination due to long-term practicing (Dong et al., 2012). Furthermore, numerous studies have reported that visual areas respond comparably to tactile, olfactory and auditory processing after long-term training or adaption (Garey, 1984; Collignon et al., 2013; Anurova et al., 2015). Therefore, the decreased regional spontaneous neural activity of the visual cortex in professional Pingju opera actors might contribute to other non-visual functions in addition to improving visual ability, which requires confirmation in future studies.

These alterations in ALFF and ReHo activity in professional traditional Chinese Pingju actors may be caused by long-term professional training, their innate predisposition, or both. In this study, we did not identify any significant correlations between regional brain activity and the duration of opera training, indicating that natural (talent) rather than nutritional (training) factors might better describe the brain changes seen in professional traditional Chinese Pingju opera actors. However, this finding might also be caused by the relatively small sample size and the relatively homogeneous professional careers of the recruited actors in this study. Furthermore, another limitation was that the behavioral assessment was not performed for opera actors and controls; thus the exact mechanism of altered brain ALFF and ReHo activities in professional Pingju opera actors remains unclear. Therefore, future studies should focus on these issues by recruiting larger sample sizes with different professional levels and performing a full performance assessment.

In summary, we observed significantly lower spontaneous regional brain activity in the visual cortex and higher brain activity in the anterior insula cortex in professional traditional Pingju opera actors, which might indicate superior performance in multidimensional professional skills, such as music and face perception, dancing, and emotional representation.

## Author Contributions

LM designed the experiments. WZ wrote the manuscript for this research. FZ scanned all the volunteers. WQ analyzed the imaging data.

## Conflict of Interest Statement

The authors declare that the research was conducted in the absence of any commercial or financial relationships that could be construed as a potential conflict of interest.

## Footnotes

1 http://www.fil.ion.ucl.ac.uk/spm

2 http://www.rfmri.org/

## Abbreviations

ALFF: amplitude of low frequency fluctuation
BOLD: blood oxygen level-dependent
FC: functional connectivity
FDR: false discovery rate
FFT: fast Fourier transform
FOV: field of view
FWHM: full-width at half maximum
MNI: Montreal Neurological Institute
PET: positron emission tomography
ReHo: regional homogeneity
Resting-state fMRI: resting-state functional magnetic resonance imaging
SPM8: statistical parametric mapping 8
TE: echo time
TI: inversion time
TR: repetition time
